# Postbiotic derived from *Bacillus subtilis* ACCC 11025 improves growth performance, mortality rate, immunity, and tibia health in broiler chicks

**DOI:** 10.3389/fvets.2024.1414767

**Published:** 2024-07-19

**Authors:** Desheng Li, Shan Fang, Feng He, Xinyan Fan, Tieliang Wang, Zeliang Chen, Mi Wang

**Affiliations:** ^1^College of Husbandry & Veterinary Medicine, Jinzhou Medical University, Jinzhou, China; ^2^Key Laboratory of Animal Product Quality and Safety of Liaoning Province, Jinzhou Medical University, Jinzhou, China; ^3^College of Animal Science and Technology, Jilin Agricultural University, Changchun, China; ^4^Liaoning Kaiwei Biotechnology Co., Ltd., Jinzhou, China

**Keywords:** small intestine health, immunity, tibia characteristic, survival rate, killed probiotic

## Abstract

**Introduction:**

The objective of this study was to evaluate the effects of dietary supplementation of postbiotics on growth performance, mortality rate, immunity, small intestinal health, tibia characteristics, and hematological parameters of broiler chicks. he postbiotics were derived from *Bacillus subtilis* ACCC 11025.

**Methods:**

A total of 480 day-old Arbor acre broiler chicks (52.83 ± 1.38 g) were used in a 42-day study and were randomly allocated into four groups. Each group comprised 6 replicate cages, each containing 20 birds. Dietary treatments were based on a basal diet, supplemented with postbiotics at concentrations of 0.000%, 0.015%, 0.030%, or 0.045%.

**Results and discussion:**

The results demonstrated an improvement in growth performance, antibody titers against avian influenza virus and Newcastle disease virus, serum albumin levels, and serum total protein levels, as well as a reduction in mortality rate among broiler chicks with increasing levels of postbiotic supplementation. The most significant effect were observed in the group receiving 0.015% postbiotics. Furthermore, a dose-dependent enhancement in tibia weight and tibia weight to length ratio, coupled with a reduction in the robusticity index, was noted. The most favorable outcomes for tibia health were observed in the group receiving 0.030% postbiotics. This improvement in tibia health corresponded to a linear increase in serum calcium and inorganic phosphorus contents. In summary, supplementing broiler chicks with 0.015% postbiotics effectively enhances immunity, leading to improved growth performance and reduced mortality rates. Additionally, a postbiotic dose of 0.030% is suitable for optimizing tibia health.

## Introduction

For an extended period, the poultry industry has benefited from antibiotic supplementation, which has been instrumental in promoting growth, enhancing intestinal health, and bolstering immunity (1). However, the detrimental effects associated with antibiotic use have gained widespread recognition (2). Consequently, in the context of antibiotic-free poultry husbandry, the industry has faced significant challenges characterized by elevated mortality rates and suboptimal growth (3). High mortality rates and poor growth performance in broiler chicks are closely linked to their immune status, intestinal health, and tibia health (4–6).

Broiler chicks are highly susceptible to various diseases due to their underdeveloped immune system. Notably, the immune system of broiler chicks does not reach maturity until around day 30 to 34 post-hatch (7). Establishing a robust and healthy gut plays a pivotal role in optimizing the growth performance and immunity of poultry. The small intestine is central to nutrient absorption and maintaining overall gut health in chicks (8, 9). Furthermore, tibia health is crucial for their overall growth and well-being, directly impacting their leg health and locomotion (10). To secure favorable body weight and survival rates among broiler chicks, it is imperative to implement measures that enhance their immune function, small intestine health, and tibia health.

In the quest for antibiotic alternatives, probiotic supplementation has emerged as an effective strategy to enhance immunity (11), support tibia health (12), maintain intestinal health (13), reduce mortality rates (14), and improve growth performance (15). It is crucial to highlight that the beneficial effects of probiotics are not solely contingent upon their viability (16). Research has shown that non-viable microorganisms, including inactive bacteria, can also confer benefits in terms of poultry growth and production (17, 18). These non-viable microorganisms are commonly referred to as “postbiotics.”

According to the International Scientific Association of Probiotics and Prebiotics in 2021, a postbiotic is defined as a “preparation of inanimate microorganisms and/or their components that confers a health benefit on the host” (19). A postbiotic should derive from microorganisms, does not have to be derived from probiotics, and should undergo a process to terminate cell viability. The final postbiotic must contain inactivated microbial cells or cell components, with viable cells absent or negligible in the final product (19, 20).

*Bacillus subtilis*, a well-established probiotic, has been extensively used in poultry diets, yielding benefits such as improved growth performance through modulation of intestinal microbiota (21), immune system regulation (22), and heightened disease resistance (23). However, the exploration of postbiotics derived from *Bacillus subtilis* remains relatively unexplored. While research on the effects of *Bacillus subtilis*-derived postbiotics in poultry is still limited, promising insights have emerged from studies such as those published by Zhu et al. (24). Their findings demonstrated that dietary supplementation with heat-inactivated *Bacillus subtilis* and *Lactobacillus acidophilus* BFI at a 1:1 ratio effectively enhanced the growth performance of native broiler chicks by influencing the composition of intestinal microbiota.

Building upon these promising preliminary findings, our research endeavors aim to explore the influence of dietary supplementation with postbiotics derived from *Bacillus subtilis* on growth performance, mortality rate, hematological parameters, small intestine health, tibia health, and immunity of broiler chicks. Our working hypothesized that this intervention will improve growth performance and reduce mortality rates through enhancing immunity, small intestine health, and tibia health.

## Materials and methods

### Experimental design

A total of 480 day-old (as hatched) Arbor Acre broiler chicks (52.83 ± 1.38 g) were used in a 42-day study and were randomly allocated into four groups. Each group comprised 6 replicate cages, each containing 20 birds. Dietary treatments were based on a basal diet, supplemented with postbiotics at concentrations of 0.000, 0.015, 0.030%, or 0.045%. The postbiotics were derived from *Bacillus subtilis* ACCC 11025, obtained from Jiangsu Yinong Biological Co., Ltd. (Jiangsu, China). According to the manufacture, *Bacillus subtilis* ACCC 11025 with 1 × 10^9^ colony-forming units per gram were inactivated by high temperature and then spray-dried to prepare the final product. The experimental protocol and all associated procedures were executed with stringent ethical standards, receiving the requisite approval and oversight from the Animal Care and Use Committee of Jinzhou Medical University (Jinzhou, China).

Birds were housed in three-tier battery cages, each measuring 1.25 meters in length, 0.80 meters in width, and 0.50 meters in height. These cages were situated within a carefully controlled experimental environment, where ambient temperature conditions were systematically managed. The temperature regimen commenced at 33°C and was subsequently decreased by 3°C on a weekly basis until it stabilized at 22°C. Concurrently, the relative humidity within the facility was consistently maintained at 65%.

Throughout the experiment, birds had uninterrupted access to both feed and water. The formulation of diets was predicated on the optimization of nutritional requirements derived from the recommendations put forth by the National Research Council. This formulation has been effectively employed in commercial practices (Boin Feed Company, Shenyang, China). The specific details of the diets are outlined in Table 1.

**Table 1:** Composition and nutrient levels of the experimental basal diet, (%, as-fed basis).

Ingredients, %	Days 1–12	Days 22–42
Corn	58.92	58.40
Soybean meal	31.00	27.10
Cottonseed meal	1.00	1.00
Corn flour	4.00	3.70
Limestone	1.00	0.90
Calcium bicarbonate	1.00	0.90
NaCl	0.25	0.25
Sodium humate	0.10	0.10
Mineral and vitamin mixture^a^	0.30	0.30
Lysine	0.75	0.74
Methionine	0.30	0.25
Threonine	0.14	0.12
Choline chloride	0.12	0.12
Betaine	0.02	0.02
Soy oil	1.00	6.00
Baking soda	0.10	0.10
Total	100.00	100.00
Analyzed composition, %		
Metabolizable energy, MJ/kg	12.13	12.76
Crude protein	21.25	20.00
Calcium	0.70	0.60
Available phosphorus	0.31	0.31
Total phosphorus	0.53	0.50
Lysine	1.41	1.35
Methionine	0.60	0.65
Methionine + Cysteine	0.94	0.99

a Provided per kilogram of diet: 1,500 IU retinyl acetate, 3,200 IU cholecalciferol; 10 IU _DL_-tocopheryl acetate; 0.5 mg menadione sodium bisulfite; 1.8 mg thiamin mononitrate; 3.6 mg riboflavin; 3.5 mg pyridoxine hydrochloride; 0.01 mg cyanocobalamin; 0.15 mg biotin; 0.55 mg folic acid; 30 mg nicotinic acid; 10 mg pantothenic acid; 8 mg copper; 0.35 mg iodine; 80 mg iron; 60 mg manganese; 0.15 mg selenium; 40 mg zinc.

### Sampling and measurements

#### Evaluating growth performance

An electronic scale was used to measure the body weight of broiler chicks on days 1, 21 and 42 of the study. Fresh feed was provided to the broiler chicks every morning, with the amount provided weighed in advance. The feed was changed the next morning, and the remaining feed was weighed. Feed intake (FI) was calculated as the difference between the feed given and the remaining amount. Data on body weight were used to calculate the average daily gain (ADG) using the following formula:


ADG=finalbodyweight−initialbodyweightexperimentalperiod×100%

Data on ADG and FI were used to calculate the feed conversion ratio (FCR) using the following formula:


FCR=FIADG×100%

#### Evaluating mortality rate

The count of dead birds was recorded daily to calculate the mortality rate for days 1–21, 22–42, and 1–42 using the following formula:


Mortalityrate=NumberofdeadbirdsInitialnumberofbirds×100%

#### Measuring antibody titer and hematological parameters

A total of eighteen birds were randomly selected from each group (3 birds per replicate cage) for blood sampling on days 21 and 42. Blood samples were collected from the wing vein and subsequently subjected to centrifugation at 3,000 × *g* at 4°C for 15 min to isolate the serum. Hemagglutination inhibition (HI) tests were used to evaluate the antibodies against avian influenza (AI; subtype H9) and Newcastle disease virus (NDV) (25). Colorimetric methods were employed to measure the concentrations of total protein and albumin in the serum (26). Globulin levels were calculated as the difference between the total protein and albumin. A fully automated biochemical analyzer (SMT-120VP, Seamaty, Chengdu, China) was used to assess the concentration of aspartate aminotransferase, alanine aminotransferase, alkaline phosphatase, creatinine, blood urea nitrogen, calcium, and inorganic phosphorus.

#### Evaluating the relative weight of immune organ

Following the blood sampling procedure, the birds were euthanized by administering 1 mL of Euthasol^®^ intravenously. Subsequently, immune organs such as the spleen, bursa of Fabricius, and thymus were excised to determine the relative organ weights using the following formula:


Organindex=organweightcarcassbodyweight×100%

#### Assessing intestinal health

In a similar manner, the duodenum, jejunum, and ileum were excised using the aforementioned methods, and the relative weight for each segment was calculated using the same formula. Subsequently, the length of the duodenum, jejunum, and ileum was measured using a ruler to assess their physical dimensions. Furthermore, the assessment of reddish lesions was conducted using a 4-point scoring system ranging from 0 to 3. In this scoring system, a lesion scored as 1 indicated the presence of one or two clearly visible reddish lesions on the intestinal segment. A lesion score of 3 was assigned when large reddish lesions were observed at multiple sites within the intestinal segments. Lesions scored as 2 exhibited an intermediate appearance between those scored as 1 or 3 (27).

#### Evaluating tibia health

The tibia bones of euthanized bird were extracted with drumsticks intact, boiled for 10 min, and subsequently defleshed and air-dried for 24 h. Measurements included tibia length, weight, thickness of medial and lateral walls, and medullary canal diameter at the diaphysis midpoint. The tibia weight to length ratio was calculated to assess bone density. Additionally, the tibiotarsal (28) and robusticity (29) indexes were calculated to assess structural strength and robustness of the bones using the following formulas, respectively:


Tibiotarsalindex=diaphysisdiameter−medullarycanaldiameterdiaphysisdiameter×100

Robusticityindex=tibialengthcuberootoftibiaweight

### Statistical analysis

In this study, the replicate cage served as the experimental unit. All collected data underwent the Kolmogorov–Smirnov test to assess normal distribution. Data showing normal distribution were analyze for linear and quadratic effects using the MIXED Model procedure of SAS Institute Inc. (Cary, NC, United States). Tukey’s test was employed to analyze the differences in data showing normal distribution. Data that did not exhibit normal distribution (mortality rate and intestinal reddish lesions) were analyzed using the nonparametric Kruskal-Wallis test. Variability within the dataset was expressed as the standard error of means, and statistical significance was defined as *p* < 0.05.

## Results

### Growth performance

**Figure 1:**
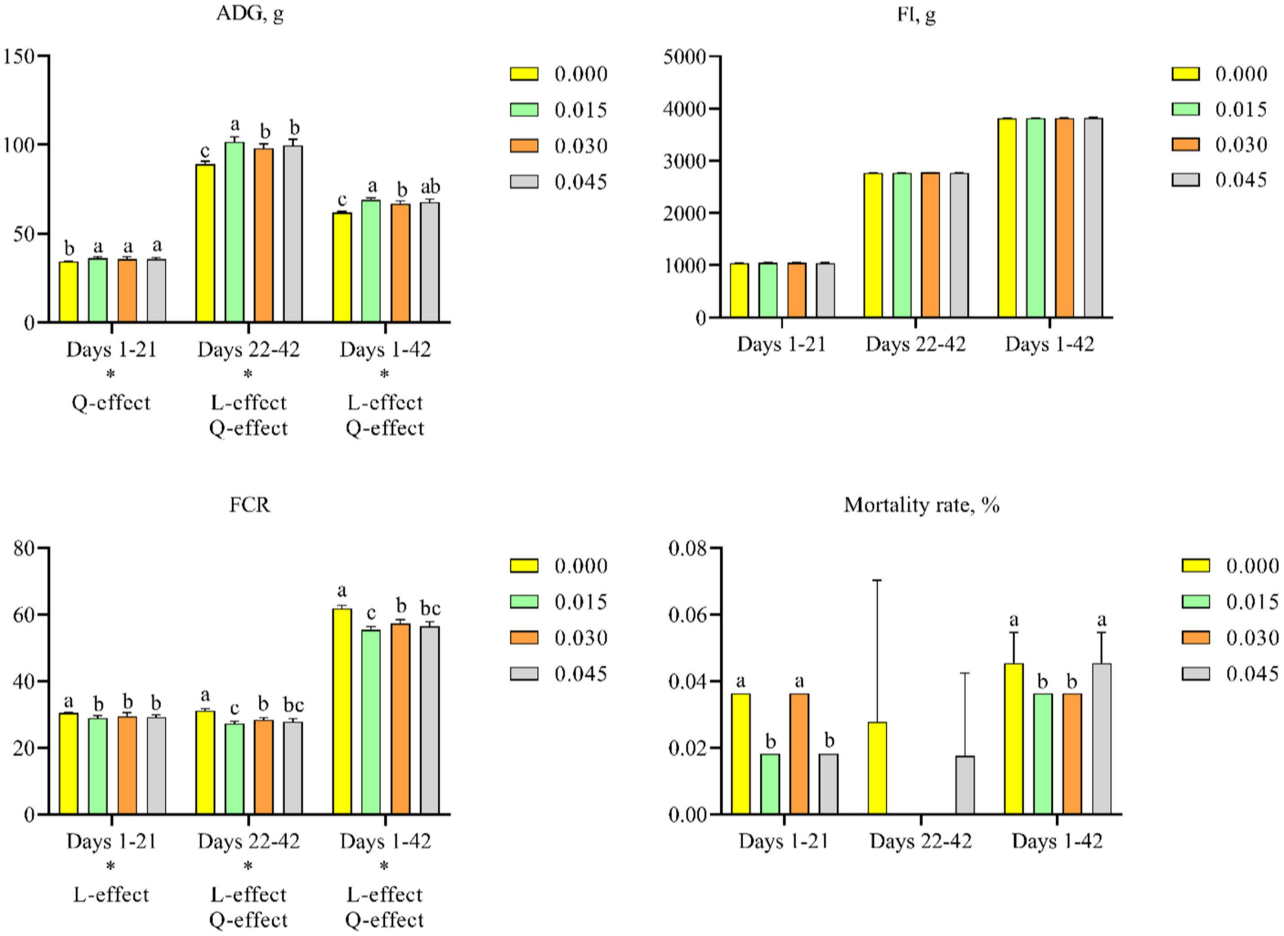
Effects of dietary supplementation with postbiotics on growth performance and mortality rates of broiler chicks. Values represent the means of 6 replicates per group (*n* = 6). ^a–c^Columns in the same parameter with different superscript differ significantly (*p* < 0.05). L-effect means the parameter has a linear effect as the concentration increases. Q-effect means the parameter has a quadratic effect as the concentration increases. ADG, average daily gain; FI, feed intake; FCR, feed conversion ratio.

Supplementing the diet of broiler chicks with postbiotics resulted in dose-dependent improvements in ADG and decreased FCR. Specifically, postbiotic supplementation quadratically increased ADG on days 1–21 (*p* < 0.05), days 22–42 (*p* < 0.05), and days 1–42 (*p* < 0.05), and linearly on days 22–42 (*p* < 0.05) and 1–42 (*p* < 0.05). Comparative analysis among groups indicated positive effects on ADG across all postbiotic doses, with the most significant improvement observed at 0.015% supplementation (*p* < 0.05) (Figure 1A). Additionally, FCR linearly decreased on days 1–21 (*p* < 0.05), days 22–42 (*p* < 0.05), and days 1–42 (*p* < 0.05), and quadratically on days 22–42 (*p* < 0.05) and 1–42 (*p* < 0.05). Notably, the 0.015% postbiotic group showed the lowest FCR (*p* < 0.05) (Figure 1C). However, postbiotics supplementation did not significantly affect FI (Figure 1B).

A significant time effect was observed for ADG (*p* < 0.05), FI (*p* < 0.05), and FCR (*p* < 0.05). Moreover, ADG (*p* < 0.05) and FCR (*p* < 0.05) were significantly affected by postbiotic supplementation (Figure 1). An interaction between treatment and time was also observed for ADG (*p* < 0.05) and FCR (*p* < 0.05) (Table 2).

**Table 2:** Effects of dietary supplementation with postbiotics on growth performance of broiler chicks at different timepoints.^a^

Items	Postbiotics, %					*p*-value		
	0.000	0.015	0.030	0.045	SEM	Time	Treatment	Treatment × Time
ADG, g	–	–	–	–	0.799	<0.001	<0.001	<0.001
FI, g	–	–	–	–	2.443	<0.001	0.180	0.811
FCR	–	–	–	–	0.391	<0.001	<0.001	<0.001

ADG, average daily gain; FI, feed intake; FCR, feed conversion ratio; SEM, standard error of the mean.

a Values represent the means of 6 replicates per group (*n* = 6).

### Mortality rate

Feeding broiler chicks with postbiotic-contained diet significantly decreased mortality rate. Specifically, supplementation with 0.015% postbiotics significantly decreased mortality rate on days 1–21 (*p* < 0.05) and days 1–42 (*p* < 0.05). Additionally, reductions in mortality rate were observed on days 1–21 with 0.045% postbiotics supplementation (*p* < 0.05), and on days 1–42 with 0.030% postbiotics supplementation (*p* < 0.05) (Figure 1D).

### Antibody titer

**Figure 2:**
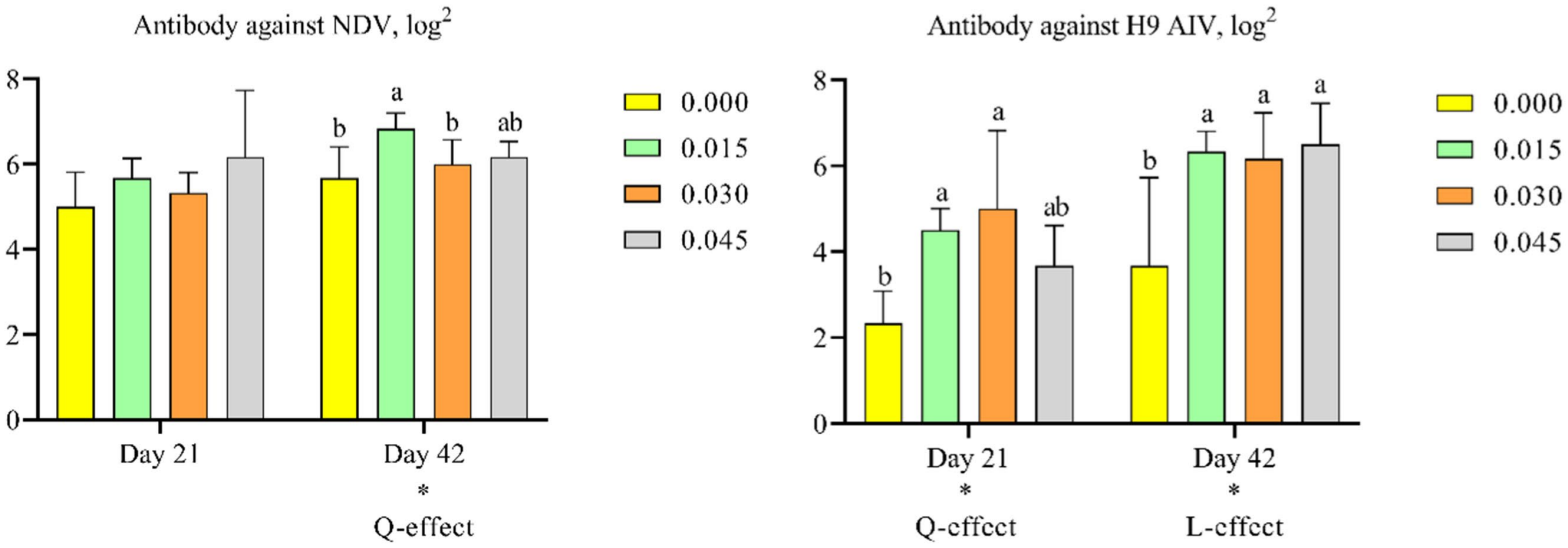
Effect of dietary supplementation with postbiotics on antibody titers of broiler chicks. Values represent the means of 6 replicates per group (*n* = 6). ^a,b^Columns in the same parameter with different superscript differ significantly (*p* < 0.05). L-effect means the parameter has a linear effect as the concentration increases. Q-effect means the parameter has a quadratic effect as the concentration increases. AIV, avian influenza virus; NDV, newcastle disease virus.

Supplementation with postbiotics linearly increased antibody levels against H9 AIV on day 42 (*p* < 0.05), and quadratically increased antibody levels against H9 AIV on day 21 (*p* < 0.05) and NDV on day 42 (*p* < 0.05). Post-hoc analysis revealed that only the 0.015% postbiotic dose significantly increased antibody levels against NDV on day 42 (*p* < 0.05). For H9 AIV antibodies, both the 0.015 and 0.030% doses were effective in increasing antibody levels on days 21 (*p* < 0.05) and 42 (*p* < 0.05) (Figures 2A,B).

### Immune organ

**Figure 3:**
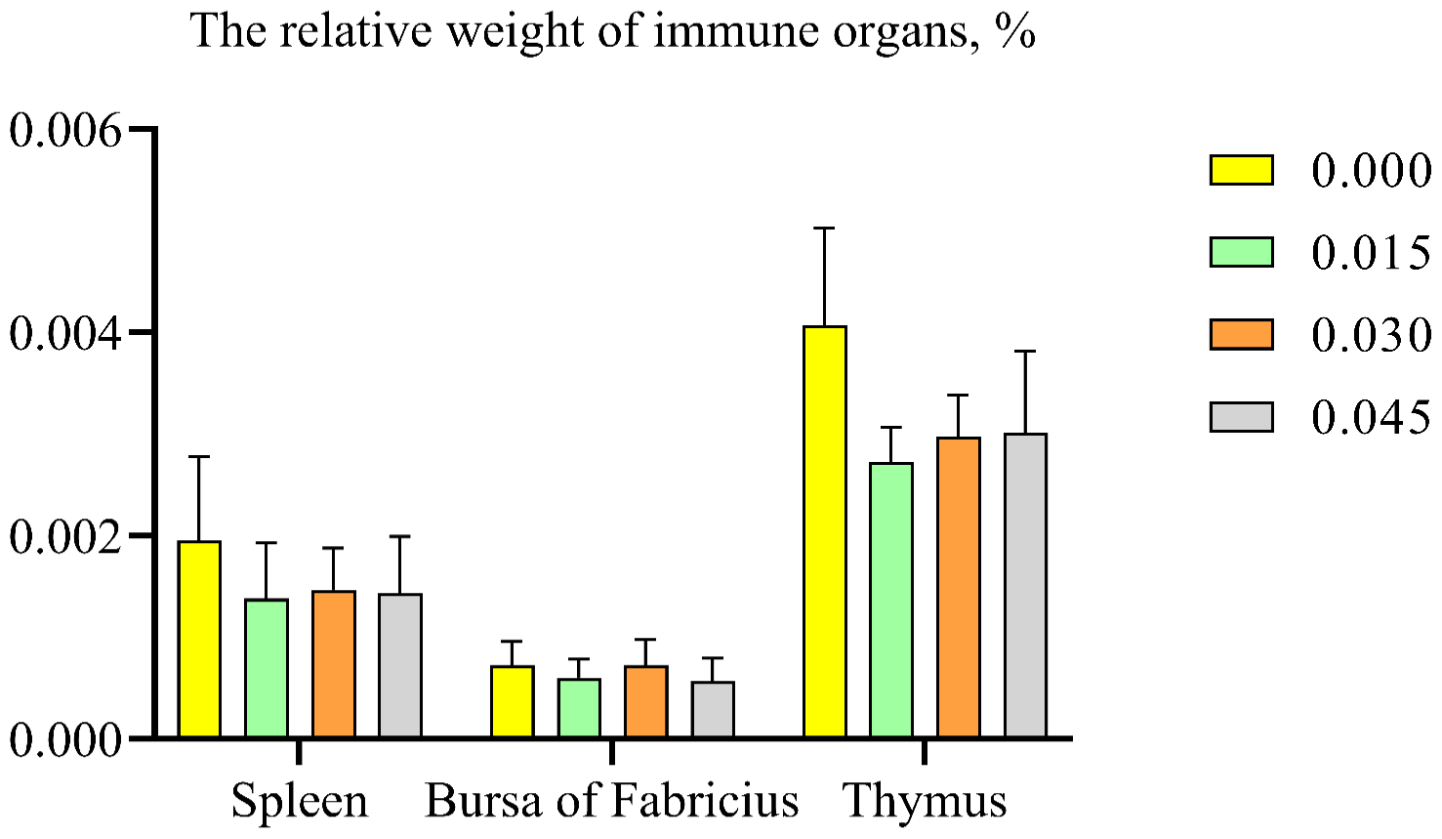
Effect of dietary supplementation with postbiotics on the relative weight of immune organs of broiler chicks. Values represent the means of 6 replicates per group (*n* = 6).

No significant differences were observed in the immune organ index, including spleen, bursa of Fabricius, and thymus, among the various groups (Figure 3).

### Intestinal health

**Figure 4:**
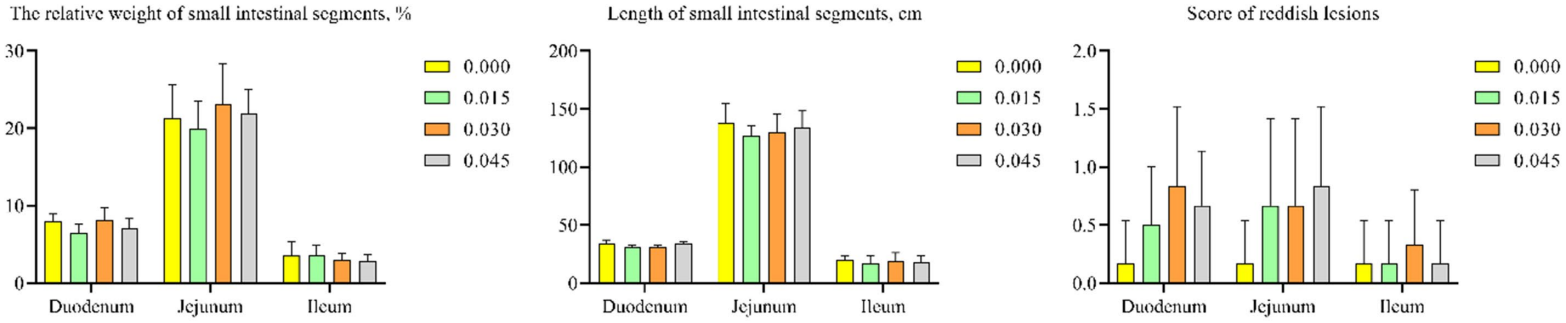
Effect of dietary supplementation with postbiotics on small intestinal health parameters of broiler chicks. Values represent the means of 6 replicates per group (*n* = 6).

Postbiotic supplementation did not significantly affect the relative weight or length of small intestinal segments. Additionally, scores for reddish lesions in the intestines did not differ notably among the groups (Figures 4A–C).

### Tibia health

**Figure 5:**
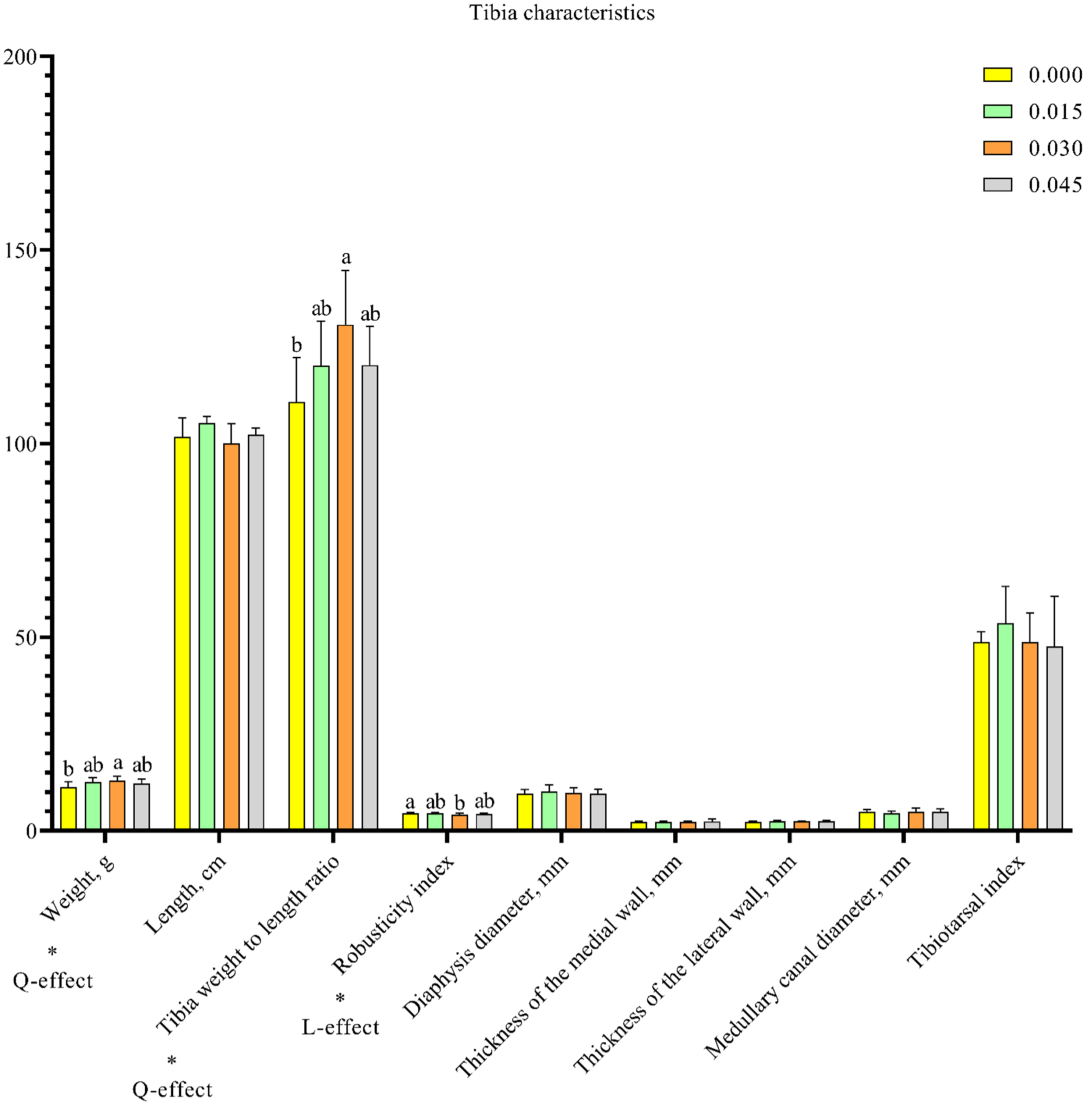
Effect of dietary supplementation with postbiotics on tibia characteristics of broiler chicks. Values represent the means of 6 replicates per group (*n* = 6). ^a,b^Columns in the same parameter with different superscript differ significantly (*p* < 0.05). L-effect means the parameter has a linear effect as the concentration increases. Q-effect means the parameter has a quadratic effect as the concentration increases.

Parameters of tibia health, including tibia weight (*p* < 0.05) and tibia weight to length ratio (*p* < 0.05), increased quadratically, while the robusticity index (*p* < 0.05) decreased linearly with postbiotic supplementation in the diet (Figure 5). Notably, significant effects on these parameters were observed only with the 0.030% dose.

### Hematological parameters

**Figure 6:**
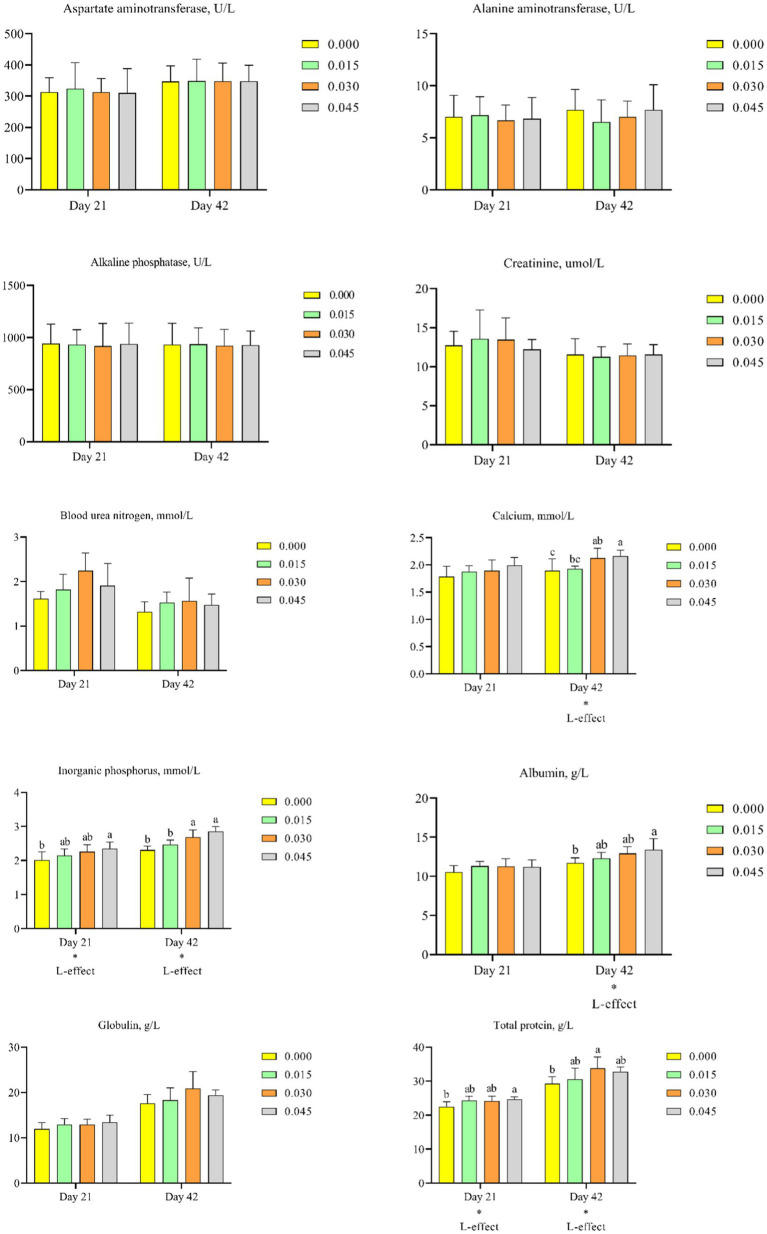
Effect of dietary supplementation with postbiotics on hematological parameters of broiler chicks. Values represent the means of 6 replicates per group (*n* = 6). ^a,b^Columns in the same parameter with different superscript differ significantly (*p* < 0.05). L-effect means the parameter has a linear effect as the concentration increases.

Consumption of postbiotics did not significantly affect parameters such as aspartate aminotransferase (Figure 6A), alanine aminotransferase (Figure 6B), alkaline phosphatase (Figure 6C), creatinine (Figure 6D), blood urea nitrogen (Figure 6E), and globulin (Figure 6I). However, postbiotics supplementation linearly increased the concentration of calcium on day 42 (*p* < 0.05; Figure 6F), inorganic phosphorus on days 21 (*p* < 0.05) and 42 (*p* < 0.05; Figure 6G), albumin on day 42 (*p* < 0.05; Figure 6H), and total protein on days 21 (*p* < 0.05) and 42 (*p* < 0.05; Figure 6J).

Comparative analysis revealed that the highest calcium concentration on day 42 was observed in the 0.030 and 0.045% postbiotics groups (*p* < 0.05). The highest inorganic phosphorus concentration on day 21 was observed in the 0.045% group, and on day 42 in the 0.030 and 0.045% groups (*p* < 0.05). The highest albumin concentration on day 42 was observed in the 0.045% group (*p* < 0.05). The highest total protein concentration on day 21 was observed in the 0.045% group, and on day 42 in the 0.030% group (*p* < 0.05).

## Discussion

This study highlights the enhanced growth performance of broiler chicks through graded supplementation with postbiotic. These fundings are consistent with previous research, emphasizing the positive impact of such supplementation on broiler performance. For instance, Incharoen et al. (30) demonstrated that dietary supplementation with heat-killed *Lactobacillus plantarum* L-137 effectively improved growth performance in broiler chicks. Similarly, Khonyoung and Yamauchi (31) found that supplementation with heat-killed *Lactobacillus sakei* HS-1 increased ADG and decreased FCR in broiler chicks. Growth performance serves as an indirect indicator of broiler health, healthy growth and achieving target weights typically indicate good health. Conversely, slow growth patterns may indicate underlying health issues (32). Thus, the observed benefits of dietary postbiotic supplementation on growth performance suggest an improvement of overall health and well-being in broiler chicks.

Assessment of serum biochemical parameters, such as albumin, globulin, and total protein, provides valuable insights into the *in vivo* health status of animals (33). Circulating total protein levels are typically lower in diseased broiler chicks compared to healthy birds (34). In the current study, a linear increase in serum albumin and total protein content was observed in animals treated with postbiotics. Therefore, dietary supplementation with postbiotics was beneficially impact the overall health of birds, as indicated by increased levels of albumin and total protein in their serum. These findings align with the concept that healthier birds tend to exhibit higher levels of these serum proteins, reflecting improved health and well-being.

Indeed, there is a correlation between serum total protein, albumin, globulin, and the antibody response indicative of immunity in broiler chicks (35, 36). Antibody titers serve as indicators of antibody levels in the bloodstream. These antibodies are proteins produced by the immune system in response to exposure to pathogens such as viruses or bacteria. Elevated antibody titers indicate that the chick has encountered a specific pathogen and mounted an immune response against it. This immune response provides protection against potential future infections caused by the same pathogen. In the present study, dietary supplementation with postbiotics resulted in increased antibody titers in broiler chicks. This observation is consistent with Nofouzi et al. (18), who reported increased antibody titers against NDV and AIV in broiler chicks following administration of heat-killed *Tsukamurella inchonensis*. Therefore, it is reasonable to conclude that postbiotic supplementation exerts positive effects on the immunity of broiler chicks, as evidenced by enhanced antibody titers.

Immune organs such as the spleen, bursa of Fabricius, and thymus are crucial for the immunological function of broiler chicks. A reduction in the weight of these organs may indicate immunosuppression, while an increase suggests enhanced immune function (37). In the present study, dietary supplementation with postbiotics did not significant affect the weight of these immune organs. This finding is consistent with Incharoen et al. (30), who found that dietary supplementation with heat-killed *Lactobacillus plantarum* L-137 did not affect the spleen index. Similarly, Zhu et al. (24) observed no changes in the relative weight of the bursa of Fabricius and spleen following dietary supplementation with a compound probiotic of heat-inactivated *Bacillus subtilis* and *Lactobacillus acidophilus* BFI. Thus, it can be hypothesized that dietary supplementation with postbiotics may not significantly enhance the immunity of broiler chicks through alterations in immune organ indices.

The small intestine plays a vital role in nutrient absorption and immune function, making its health crucial for optimal growth and immunity. The relative weight and length of small intestinal segments can indicate the development of specific segments within the small intestine (38). However, in the current study, dietary supplementation with postbiotics did not significant affect the development of small intestinal segments. This finding contrasts with Khonyoung and Yamauchi (31), who reported decreased weights of the ileum, total small intestine, and ceca with increasing supplementation of heat-killed *Lactobacillus sakei* HS-1 in broiler chicks. Therefore, different postbiotic types may generate different effects on intestinal health.

Additionally, scoring reddish lesions in the small intestine is typically used to assess the presence and severity of lesions associated with conditions like coccidiosis or *Clostridium perfringens* infection, which can cause small red petechiae in the mid-intestine (39, 40). These lesions are closely related to high intestinal fragility (41). In the present study, supplementation with postbiotics did not significantly impact the score of reddish lesions, possibly due to the absence of coccidiosis or *Clostridium perfringens* infection within the experimental block.

Tibia health is crucial for poultry, directly impacting their overall well-being. The structural support provided by the tibia allows poultry to engage in various activities such as standing, walking, and perching. Poultry with strong tibia are better equipped to resist diseases and infections, while weakened or fragile tibia can render birds more susceptible to injuries and infections (42). In the current study, dietary supplementation with postbiotics positively affected tibia health parameters, including tibia weight and tibia weight to length ratio, while it negatively affected the robusticity index. The tibia weight to length ratio is a straightforward indicator of bone density, with higher values indicating denser bone structures (43). Similarly, higher tibia weight values indicate greater bone mineralization (44). A lower robusticity index suggests a robust bone structure (45). Calcium and phosphorus are essential minerals for bone mineralization in broilers, and their deficiency can impair bone development and related metabolic utilization parameters (46, 47). In the present study, dietary postbiotic supplementation led to increased serum calcium and phosphorus contents. As reported by Li et al. (47), broiler chicks with higher calcium and phosphorus levels in their serum tend to exhibit better bone quality. Therefore, it is reasonable to conclude that dietary supplementation with postbiotics can enhance tibia health.

Alkaline phosphatase, aspartate aminotransferase, and alanine aminotransferase are parameters that provide insight into liver health (48). An increase in their serum levels may indicate liver damage (48). Additionally, creatinine and blood urea nitrogen are indicators of renal function (49). Elevated levels in serum are typically associated with kidney injury (50). In the present study, dietary supplementation with postbiotics did not significantly affect alkaline phosphatase, aspartate aminotransferase, alanine aminotransferase, creatinine, and blood urea nitrogen levels. These observations suggest that postbiotic supplementation did not adversely affect liver or renal function in broiler chicks.

Enhancing the health condition of broiler chicks can positively impact their survival rate. In the present study, dietary supplementation with postbiotics improved immunity and tibia health in broiler chicks. This is consistent with findings from previous research. Wu et al. (51) reported that enhancing immunity effectively reduces mortality caused by *Escherichia coli* infection in broiler chicks. Furthermore, Liu et al. (52) found that supplementation with *Lactobacillus rhamnosus* improved normal growth and development of the tibia growth plate, thereby protecting broiler survival rates. Based on these findings, it can be speculated that dietary supplementation with postbiotics enhances immunity and tibia quality in broiler chicks, contributing to a reduced mortality rate.

## Conclusion

In conclusion, this research highlights the potential benefits of dietary supplementation with postbiotics derived from *Bacillus subtilis* ACCC 11025 in broiler chick nutrition. This postbiotic have demonstrated the capacity to enhance growth performance, reduce mortality rates, boost immunity, and improve tibia health without compromising liver or renal function. These findings contribute to the development of effective and sustainable poultry farming practices essential for the growth and well-being of the poultry industry.

## Data availability statement

The raw data supporting the conclusions of this article will be made available by the authors, without undue reservation.

## Ethics statement

The animal study was approved by the experimental protocol and all associated procedures were executed with stringent ethical standards. Additionally, it received the requisite approval and oversight from the Animal Care and Use Committee of Jinzhou Medical University (Jinzhou, China). The study was conducted in accordance with the local legislation and institutional requirements.

## Author contributions

DL: Investigation, Writing – original draft, Writing – review & editing. SF: Conceptualization, Data curation, Investigation, Methodology, Writing – original draft. FH: Conceptualization, Data curation, Investigation, Methodology, Writing – original draft. XF: Conceptualization, Data curation, Investigation, Methodology, Writing – original draft. TW: Conceptualization, Data curation, Investigation, Methodology, Writing – original draft. ZC: Conceptualization, Data curation, Investigation, Methodology, Writing – original draft. MW: Supervision, Writing – review & editing.
